# Dosing strategy of intrapleural bortezomib for myelomatous pleural effusion: A case report and review of literature

**DOI:** 10.1002/jha2.998

**Published:** 2024-10-10

**Authors:** Yu‐Ming Li, Mei‐Hua Tsou, Tran‐Der Tan

**Affiliations:** ^1^ Department of Internal Medicine Koo Foundation Sun Yat‐Sen Cancer Center Taipei Taiwan; ^2^ Department of Pathology and Laboratory Medicine Koo Foundation Sun Yat‐Sen Cancer Center Taipei Taiwan; ^3^ Department of Hematopoietic Stem Cell Transplantation and Cell Therapy Koo Foundation Sun Yat‐Sen Cancer Center Taipei Taiwan; ^4^ Present address: Department of Integrative Immunobiology Duke University School of Medicine Durham North Carolina USA

**Keywords:** bortezomib, case report, multiple myeloma, pleural effusion

## Abstract

Myelomatous pleural effusion (MPE) is a rare, often treatment‐resistant complication of multiple myeloma. Intrapleural bortezomib shows promise but lacks standardized dosing. We report a 62‐year‐old woman with MPE treated with 1.3 mg/m^2^ (2 mg) subcutaneous and 0.975 mg/m^2^ (1.5 mg) intrapleural bortezomib on days 1 and 4. Despite MPE regression, significant toxicity occurred. Adjusted dosing to 0.65 mg/m^2^ (1 mg) for both routes on days 11 and 14 consolidated the response without side effects. This case demonstrates the feasibility of intrapleural therapy and the importance of cautious dosing. Literature supports equal intrapleural and systemic bortezomib dosing for MPE management.

## 1 INTRODUCTION

Myelomatous pleural effusion (MPE) is characterized by myeloma cells infiltrating the pleural space, causing pleural effusion accumulation [1]. Symptoms, including shortness of breath, cough, and chest pain, vary with the effusion volume [2]. Diagnosing MPE is challenging, as it occurs in less than 1% of multiple myeloma (MM) patients [3] and can be masked by coexisting conditions like heart and renal failure [4]. A definitive diagnosis requires identifying myeloma cells in pleural fluid through cytopathology, histopathology, or flow cytometry [5]. Measuring γ globulin through fluid electrophoresis can also facilitate diagnosis [4]. Treatment primarily involves systemic chemotherapy, supplemented by drainage and pleurodesis to manage symptoms [5]. However, MPE often recurs, presumably due to poor drug delivery to the pleural space [6], leading to repeated thoracentesis and patient distress. Direct intrapleural injections of bortezomib have the potential to reduce MPE recurrence, but the dosing strategies vary across case reports [5, 6, 7, 8, 9].

We present a case of refractory MPE treated with intrapleural bortezomib combined with subcutaneous administration for one cycle. While adjusting for bortezomib toxicity, the treatment led to the successful management of MPE.

### 1.1 Case report

**FIGURE 1:**
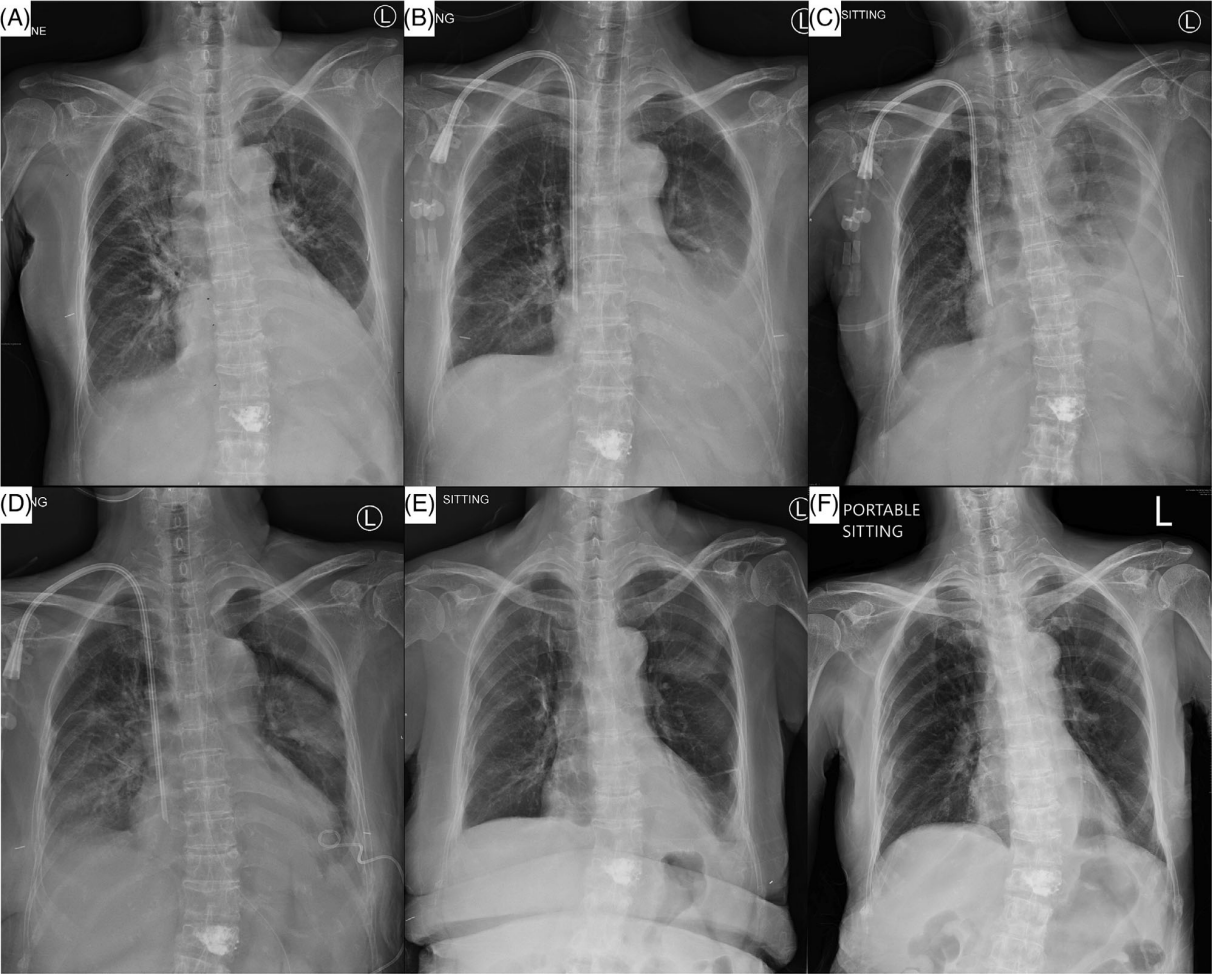
Serial chest X‐ray anterior‐posterior views showing the progression of myelomatous pleural effusion at various treatment timepoints: at the time of admission (A), 1 month following the first cycle of bortezomib, thalidomide, and dexamethasone (VTd) regimen therapy (B), 3 days before the first intrapleural injection of bortezomib (C), on the day of the final intrapleural bortezomib injection (D), at discharge (E), and 2 months after discharge (F).

A 62‐year‐old woman reported increasing fatigue for a week following vertebroplasty for an L2 compression fracture. She also had poor appetite and oliguria. Initial lab results showed leukocytosis (WBC 11,990/µL), anemia (Hb 7.7 g/dL), renal failure (creatinine 10.95 mg/dL), hyperuricemia (uric acid 11.3 mg/dL), hyperkalemia (K 5.7 mmol/L), hypercalcemia (Ca 11.5 mg/dL), hyperphosphatemia (P 7.1 mg/dL), and hypoalbuminemia (albumin 3.01 mg/dL). Signs of fluid overload, including leg edema and left pleural effusion (Figure 1A), prompted hemodialysis initiation on the fourth day after admission.

Further diagnostics revealed elevated serum IgA (1052 mg/dL) and kappa light chains (8736.5 mg/dL) with a bone marrow biopsy confirming IgA kappa MM, stage III of the International Staging System. Bortezomib, thalidomide, and dexamethasone (VTd) therapy were initiated, leading to significant decreases in serum IgA, kappa light chain, and β2‐microglobulin levels after 1 month.

**FIGURE 2:**
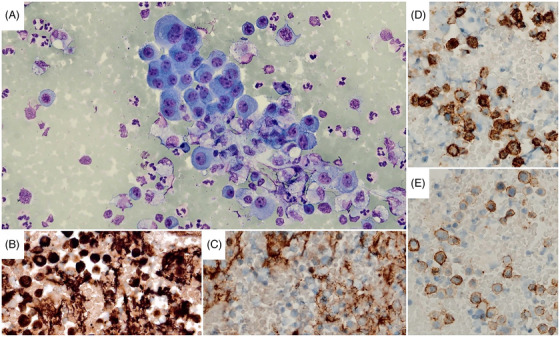
Diagnosis of myelomatous pleural effusion: (A) Cytopathological examination of left pleural effusion revealing a myeloma cell characterized by an eccentric nucleus, a “clock‐face” pattern of chromatin, and basophilic cytoplasm (Riu's stain, magnification: 200X). (B–E) Immunohistochemical staining of the myeloma cells for kappa light chain (B, magnification: 400X), lambda light chain (C, magnification: 400X), CD138 (D, magnification: 400X), and CD56 (E, magnification: 400X).

Despite systemic improvements, the patient's left pleural effusion increased, causing dyspnea (Figure 1B). Pleural fluid analysis showed a hemorrhagic exudate. Cytology and immunohistochemistry confirmed MPE (Figure 2). Recurring effusion unresponsive to systemic therapy (Figure 1C) led to the addition of intrapleural bortezomib in the third VTd cycle. Informed consent was obtained, and a 6 French chest drainage catheter was inserted for drug administration.

On days 1 and 4, the patient received 1.3 mg/m^2^ (2 mg) subcutaneous and 0.975 mg/m^2^ (1.5 mg) intrapleural bortezomib injections. After the second injection, the effusion's daily volume reduced significantly from over 1000 mL to less than 200 mL, and the dyspnea improved. However, the patient experienced common terminology criteria for adverse events (CTCAE) grade 3 watery diarrhea, grade 2 maculopapular skin rash, and grade 2 thrombocytopenia, indicating bortezomib toxicity [10]. Consequently, the dose was adjusted to 0.65 mg/m^2^ (1 mg) for both subcutaneous and intrapleural administration on days 11 and 14. No side effects were noted thereafter.

A follow‐up chest X‐ray revealed the resolution of the left pleural effusion as early as after the fourth intrapleural bortezomib injection (Figure 1D). This outcome prompted the discontinuation of intrapleural bortezomib and the removal of the drainage catheter. No pleural effusion recurrence was observed at discharge (Figure 1E) and at a 2‐month follow‐up (Figure 1F). The patient remained in complete remission for over 18 months under thalidomide 50 mg daily, continuing through to the submission of this article.

## 2 DISCUSSION

Bortezomib, a proteasome inhibitor, is widely used as a first‐line treatment for MM [11], making its intrapleural use more accessible when combined with systemic bortezomib‐based therapy for MM and MPE. Typically, bortezomib is administered into the chest cavity after draining the pleural effusion. Our protocol consisted of administering the drug through the drainage catheter, followed by flushing with 50 mL of normal saline and then clamping the tube for 2 days to retain the drug within the pleural cavity. Using an indwelling catheter for intrapleural injections allows for convenient medication delivery, reduces the need for repeated thoracentesis, and facilitates daily monitoring of the pleural effusion's volume and appearance. However, there is a risk of catheter‐associated infection, necessitating thorough education of the patient and their family on catheter care to mitigate this risk.

Our case, along with other reports [5, 6, 7, 8, 9], demonstrated the effectiveness of intrapleural bortezomib in managing MPE. Initially, we used an entire 3.5 mg ampule of bortezomib, distributing 2 mg for subcutaneous and 1.5 mg for intrapleural injections on days 1 and 4. This approach maintained the full systemic bortezomib dosage while increasing the bortezomib concentration in the pleural space. However, the resultant toxicity indicated possible systemic absorption of the drug from the pleural space, emphasizing the need for precise dosing. The adjusted dose of 1 mg for both routes on days 11 and 14 consolidated the response without further side effects.

A literature review (Table 1) revealed a trend of using at least one cycle of evenly distributed doses for systemic and intrapleural injections [5, 6, 7, 8, 9]. Notably, halving the bortezomib dose for intrapleural injections did not seem to influence the systemic response and the overall disease trajectory. Since the outcome of patients with MPE is usually poor [5], the treatment goal is recommended to help with respiratory symptom relief. Therefore, the treatment course should be tailored for each patient based on the severity of symptoms caused by the pleural effusion and their response to therapy. However, the lack of standardized dosing guidelines for intrapleural bortezomib and the absence of randomized controlled trials to evaluate its efficacy remain significant limitations. Additionally, as intrapleural bortezomib is considered off‐label use [12], thorough patient and family discussion on the benefits and risks is crucial for informed decision‐making.

**TABLE 1:** Comparison of clinical data on intrapleural bortezomib in patients with myelomatous pleural effusion between published literature and our patient.

	Patient profile	Intrapleural bortezomib dosing	Systemic bortezomib dosing	Systemic drug regimen & cycles	Total intrapleural treatments	Outcome
Iannitto et al. [6]	66 y/o male IgG‐λ MM Left‐sided MPE	0.65 mg/m^2^ (Days 1, 4, 8, 11)	0.65 mg/m^2^ (Days 1, 4, 8, 11)	Vd, two cycles	8	No recurrence of MPE, stable MM
Klanova et al. [7]	48 y/o female IgG‐κ MM Left‐sided MPE	0.75 mg/m^2^ (Days 8, 11)	0.75 mg/m^2^ (Days 8, 11)	PAd, one cycle	2	Significant MPE regression, patient died of disease progression
Sun et al. [8]	57 y/o female IgG‐κ MM Bilateral MPE	0.95 mg left side (Day 4) 0.95 mg each site (Day 8)	0.95 mg (Day 4) No bortezomib (Day 8)	DVd, one cycle	2	MPE reduction, the patient died of disease progression
Lázaro Sierra et al. [9]	65 y/o female IgA‐κ MM Left‐sided MPE	Two‐thirds (Days 1, 4, 8, 11)	One‐third (Days 1, 4, 8, 11)	VRd, two cycles	8	No recurrence of MPE
Yanamandra et al. [5]	45 y/o female Unilateral MPE	1 mg weekly (First two cycles), monthly thereafter	Not specified	CyBroD, four cycles	10	Stable condition
Yanamandra et al. [5]	69 y/o male Bilateral MPE	1 mg weekly	Not specified	VAD, two cycles	Unsure	Patient died of disease progression
Our patient	62 y/o female IgA‐κ MM Left‐sided MPE	0.975 mg/m^2^ (1.5 mg, Days 1, 4) 0.65 mg/m^2^ (1 mg, Days 11, 14)	1.3 mg/m^2^ (2 mg, Days 1, 4) 0.65 mg/m^2^ (1 mg, Days 11, 14)	VTd, one cycle	4	No recurrence of MPE, stable MM control

Abbreviations: CyBorD, cyclophosphamide, bortezomib, and dexamethasone; DVd, daratumumab, bortezomib, and dexamethasone; MM, multiple myeloma; MPE, myelomatous pleural effusion; PAd, bortezomib, doxorubicin, and dexamethasone; VAD, vincristine, doxorubicin, and dexamethasone; Vd, bortezomib and dexamethasone; VRd, bortezomib, lenalidomide, and dexamethasone; VTd, bortezomib, thalidomide, and dexamethasone; y/o, years old.

## 3 CONCLUSION

Our findings suggest that intrapleural bortezomib can be a viable option for MPE management. It warrants further research to establish standardized dosing protocols and to explore their long‐term efficacy and safety.

## AUTHOR CONTRIBUTIONS

Tran‐Der Tan conceptualized the therapeutic approach for the patient. Yu‐Ming Li and Mei‐Hua Tsou acquired and analyzed the data. Yu‐Ming Li drafted the manuscript. Mei‐Hua Tsou and Tran‐Der Tan critically revised the manuscript. All authors approved the final version of the manuscript for publication.

## CONFLICT OF INTEREST STATEMENT

The authors declare no conflict of interest.

## FUNDING INFORMATION

The authors received no specific funding for this work.

## ETHICS STATEMENT

The authors have confirmed that an ethics approval statement is not needed for this submission.

## PATIENT CONSENT STATEMENT

The written informed consent was obtained from the patient.

## CLINICAL TRIAL REGISTRATION

The authors have confirmed clinical trial registration is not needed for this submission.

## Data Availability

All data underlying the results are available as part of the article, and no additional source data are required.
